# Combination of EZH2 and ATM inhibition in BAP1-deficient mesothelioma

**DOI:** 10.1038/s41416-024-02661-3

**Published:** 2024-03-22

**Authors:** Nick Landman, Danielle Hulsman, Jitendra Badhai, Jawahar Kopparam, Julian Puppe, Gaurav Kumar Pandey, Maarten van Lohuizen

**Affiliations:** 1 Division of Molecular Genetics, The Netherlands Cancer Institute, Plesmanlaan 121, Amsterdam, The Netherlands; 2 Oncode Institute, Jaarbeursplein 6, Utrecht, The Netherlands; 3 Department of Obstetrics and Gynaecology, University Hospital of Cologne, Kerpener Str. 34, Cologne, Germany; 4 Department of Zoology, Banaras Hindu University, Varanasi, Uttar Pradesh India

**Keywords:** Mesothelioma, Mesothelioma, Preclinical research, Molecular medicine, Translational research

## Abstract

**Background:**

More than half of mesothelioma tumours show alterations in the tumour suppressor gene *BAP1*. BAP1-deficient mesothelioma is shown to be sensitive to EZH2 inhibition in preclinical settings but only showed modest efficacy in clinical trial. Adding a second inhibitor could potentially elevate EZH2i treatment efficacy while preventing acquired resistance at the same time.

**Methods:**

A focused drug synergy screen consisting of 20 drugs was performed by combining EZH2 inhibition with a panel of anti-cancer compounds in mesothelioma cell lines. The compounds used are under preclinical investigation or already used in the clinic. The synergistic potential of the combinations was assessed by using the Bliss model. To validate our findings, in vivo xenograft experiments were performed.

**Results:**

Combining EZH2i with ATMi was found to have synergistic potential against BAP1-deficient mesothelioma in our drug screen, which was validated in clonogenicity assays. Tumour growth inhibition potential was significantly increased in BAP1-deficient xenografts. In addition, we observe lower ATM levels upon depletion of BAP1 and hypothesise that this might be mediated by E2F1.

**Conclusions:**

We demonstrated the efficacy of the combination of ATM and EZH2 inhibition against BAP1-deficient mesothelioma in preclinical models, indicating the potential of this combination as a novel treatment modality using BAP1 as a biomarker.

## Background

Malignant mesothelioma (MM) is a rare and highly aggressive tumour arising from the lining of the pleural and thoracic cavity. The vast majority of MM cases can be linked to occupational asbestos exposure [1, 2]. However, due to the long latency of MM and the absence of clear symptoms during tumour onset, patients are diagnosed late in disease development. Together with limited treatment options, this leads to a poor median survival ranging between 6 and 8 months [3]. Current treatments include chemotherapy, cisplatin + pemetrexed, or the recently approved immune checkpoint blockade (ICB) therapy, nivolumab + ipilimumab [4, 5]. This novel first-line treatment with ICBs showed a statistically significant improvement in the overall survival (OS) of patients compared with those who received chemotherapy in the CHECKMATE-743 open-label trial [6, 7]. However, the median OS was only modestly improved compared to chemotherapy treatment. Together with the worldwide incidence of mesothelioma predicted to increase, more effective therapies are urgently needed [8, 9].

A better understanding of the molecular characteristics of MM has identified several molecular targets, paving the way for potential personalised therapies. However, to date there are no routinely used biomarkers in place for MM patients who are likely to respond to treatment. The genomic landscape of MM shows frequent losses of tumour suppressor genes, including frequent inactivation of the CDKN2AB (40–50% of patients) locus encoding for p14^ARF^, p15^INK4B^, p16^INK4A^ proteins and Neurofibromatosis Type 2 (NF2) gene (20–50% of patients) [10–12]. In addition, the BRCA1-associated protein 1 (BAP1) gene has been found to be mutated, deleted or epigenetically silenced in human mesothelioma [13, 14]. A study by Hmeljak and colleagues showed that the overall prevalence of BAP1 alterations in malignant mesothelioma is 57%, of which 96% were inactivating mutations [15]. The BAP1 protein is a member of the Polycomb Repressive Deubiquitinase complex (PR-DUB), where it acts as a ubiquitin carboxy-terminal hydrolase (UCH) removing ubiquitin from histone H2ALys119 [16, 17]. This deubiquitinating function of PR-DUB opposes the function of Polycomb Repressive Complexes (PRC). These two well-known complexes, PRC1 and PRC2, modify chromatin via deposition of the mainly repressive histone marks H2AK119Ub1 and H3K27me3, respectively, and are widely implicated in a multitude of malignancies [18, 19]. Interestingly, we and others have shown that BAP1-deficient MM have elevated PRC2-mediated gene repression and are vulnerable to pharmacological inhibition of EZH2, the catalytic subunit of PRC2 [20, 21].

In preclinical settings, it has been established that BAP1-deficient MM is sensitive to the inhibition of EZH2. However, results from mesothelioma mouse models show that EZH2 inhibition alone may show limited efficacy [21, 22]. In addition, the recently completed phase II multicentre trial in MM patients with inactivated BAP1 showed only modest activity upon treatment with the EZH2 inhibitor Tazemetostat, corroborating the findings from the mouse model [23]. The modest effect of EZH2 inhibitor as a single agent could suggest that complementing this treatment with an additional inhibitor could potentially improve therapy. As MM patient tumours show both spatial and temporal intra-tumour heterogeneity, in addition to higher efficacy another major benefit of the addition of a rational second drug could be the decrease in chance of developing resistance to therapy [24, 25]. Due to the important role of EZH2 in polycomb-regulated expression, it is highly likely that alterations in its expression will lead to novel dependencies. In fact, in other solid tumour types several synthetic lethal partners of EZH2 have previously been identified [26–29].

Prompted by the limited activity as a single agent in clinical trial and the potential benefits of combination therapy over monotherapy, we set out to find rational drug combinations with EZH2 inhibition. To this end, we combined the EZH2 inhibitor GSK126 with a panel of 20 existing anti-cancer compounds, that are under preclinical investigation or are already being used in the clinic, targeting prominent oncogenic signalling pathways. Here, we identified a highly synergistic potential for the combination of ATM inhibition with EZH2 inhibition in BAP1-deficient MM.

## Materials and methods

### Cell culture

Early passage murine mesothelioma cell lines were previously derived in our laboratory from autochthonous compound mesothelioma mouse models. Cells were cultured in Dulbecco’s Modified Eagle Medium/Nutrient Mixture F-12 (DMEM/F-12+Glutamax; Gibco), supplemented with 4 μg/ml Hydrocortisone (Sigma), 5 ng/ml murine EFG (Sigma), insulin-transferrin-selenium solution (ITS; Gibco), 10% foetal calf serum (FCS; Capricorn) and 1% penicillin and streptomycin (Gibco) [21, 30]. All mesothelioma cell lines derived from humans were obtained from the American Type Culture Collection (ATCC). The NCI-H226 cell line with re-expressed BAP1 was a kind gift from Prof. Sam Janes. Cell lines were cultured in mammalian cell culture medium as specified above. Uveal melanoma cell lines, also obtained from ATCC, were cultured in either Roswell Park Memorial Institute 1640 (RPMI-1640; Gibco) or Dulbecco’s Modified Eagle Medium (DMEM; Gibco) supplemented with 10% or 20% FCS and 1% penicillin/streptomycin. All cell lines were maintained at 37 °C in a humidified atmosphere containing 5% carbon dioxide (CO_2_) and were tested for mycoplasma contamination using MycoAlert Mycoplasma detection kit (Lonza). The human cell lines were authenticated using short tandem repeat STR DNA profiling.

BAP1 knock-down and knock-out cell lines were generated in our lab as previously described in Pandey and Landman et al. [22] ATM Cas9-induced knock-out cell lines were made using Alt-R CRISPR Guide RNAs (IDT DNA). A final duplex concentration of 3 μM was created by mixing crRNA and tracrRNA in equimolar concentrations. The RNP complex was formed by mixing the duplex with an equimolar amount of Alt-R spCas9 enzyme. Reverse transfection of the RNP complex was done using Lipofectamine RNAiMax (Invitrogen, product #13778075).

### Western blot analysis

Whole-cell pellets were lysed in RIPA buffer (50 mM Tris, pH 8.0, 50 mM NaCl, 1.0% NP-40, 0.5% sodium deoxycholate, and 0.1% SDS) containing protease inhibitor cocktail (Complete; Roche) and phosphate inhibitors (10 mM NaF final concentration, 1 mM Na_3_VO_4_ final concentration, 25 mM β-Glycerophosphate final concentration, 1 mM PMSF, and 1 mM Na_4_P_2_O_7_ final concentration), and 20 mM DTT. Protein concentrations were measured on a Nanodrop 2000c spectrophotometer (ThermoFisher) using Protein Assay Dye reagent (Bio-rad). Protein was loaded in equal amounts onto 4–12% Bis-Tris gels (NuPage-Novex, Invitrogen) and transferred onto nitrocellulose membranes (0.2 μm; Whatman). Membranes were blocked in 5% BSA in phosphate-buffered saline (PBS) with 0.1% Tween-20 (PBST) for 1 h, incubated with primary antibodies in PBST 1% BSA overnight at 4 °C, and incubated with secondary antibodies coupled to HRP for 45 min in PBST 1% BSA at room temperature. Amersham ECL detection reagent was used for antibody detection (GE Healthcare). Imaging of the membranes was done on a Bio-Rad ChemiDoc XRS + . The following antibodies were used for western blot analyses: BAP1 D7W70 (Cell Signalling, 13271S), p-ATM (Ser1981) (BioLegend, 651201), Tri-Methyl-Histone H3 (Lys27) C36B11 (Cell Signalling, 9733S), anti-Tubulin (Sigma, T9026).

### Drug dose response

Cell seeding densities were determined prior to dose–response experiments. Cells were counted using HyClone Trypan Blue (Cytiva) on an automated cell counter (Bio-Rad, TC20), and live cells were seeded in triplicate into 384-well plates in 50 μl of culture medium. Drug compounds, DMSO-negative control, or phenylarsine oxide (PAO) positive control were added after 24 h using the D300e digital dispenser (TECAN), and cells were grown for 72 h. Subsequently, cells were incubated for 4 h with Resazurin (Sigma), and plates were read using an Infinite M1000 pro plate reader (TECAN) with the following parameters: fluorescence, 570-nm excitation, 600-nm emission, three flashes. Results were normalised against DMSO-treated cells. Drug dose–response curves were generated with GraphPad Prism v.9 using the Nonlinear regression curve fit function using constraints for the bottom ( = 0) and top ( = 100) values.

### Synergy screen and analysis

Prior to drug synergy assays optimal seeding density of cell lines was derived from growth curves. Cells were counted with HyClone Trypan Blue (Cytiva) using a TC20 automated cell counter (Bio-Rad) and live cells were seeded into 384-well plates in 50 μl of culture medium.

Drug compounds (20 drugs in total), DMSO-negative control or PAO positive control were added after 24 h using the D300e digital dispenser (TECAN) in a 6 × 6 matrix, and cells were grown for 72 h. Drug concentrations were determined beforehand by titrating to the most sensitive cell line based on dose–response curves, exact concentration ranges used per drug are available upon request. Subsequently, cells were incubated for 4 h with Resazurin (Sigma), and plates were read using an Infinite M1000 pro plate reader (TECAN) with the following parameters: fluorescence, 570-nm excitation, 600-nm emission, three flashes. Results were normalised against DMSO-treated cells. Plate read-outs were analysed using the SynergyFinder v.2 web-based application as described by Ianevski et al. [31, 32] 3D plots, inhibition matrices and synergy scores were generated using default parameters for calculating BLISS Independence scores [33]. Heatmaps with BLISS synergy scores were generated using the geom_tile function from ggplot2 package in R.

### Colony-formation assays

Again, prior to colony-formation assay, optimal seeding densities were determined. The appropriate number of cells were seeded in 6-well culture plates and allowed to adhere overnight. Drug compound(s) or DMSO were added to cells the next day and refreshed every other day to retain stable drug concentrations. Plates were fixed after 10 days using 4% Paraformaldehyde (Merck) and stained with 0.1% crystal violet solution (Sigma) in PBS with 10% EtOH. Plates were digitised using the ChemiDoc XRS+ (Bio-Rad) and analysed using the ImageJ plugin ‘ColonyArea’ as published by Guzman et al. [34]. Representative images of three independent experiments are shown.

### Annexin V-FITC apoptotic assay

Cells were seeded in six-well culture plates and allowed to adhere overnight. Drug compounds were added the next day and incubated for 48 h. Cells were collected by centrifugation, washed once with cell culture media, centrifuged again, and resuspended in 500 μl of Annexin V binding buffer (Abcam, ab14085). In total, 5 μl Annexin V-FITC and Propidium Iodide were added and incubated in the dark for 5 min. Cells were then quantified on the Flow Cytometer (AttuneNxT, ThermoFisher) and analysed with FlowJo v10.

### RNA sequencing, analysis and GSEA

Cells were lysed in RLT buffer (Qiagen). RNA extraction, library preparation, sequencing and reads processing were performed by the Genomics Core Facility at the Netherlands Cancer Institute. Sequencing was performed using the Illumina HiSeq 2500 platform according to the standard procedures. RNA sequencing reads from mouse material were aligned to the mm10 genome with hisat2, transcript quantification was performed with HTSeq. Human samples were aligned to GRCh38 and read counts per gene using gensum. Genes were annotated using Ensembl GRCh38.102. Subsequent data analyses were performed using R and Bioconductor. DESeq2 package was used for the analysis of differential gene expression in RNA sequencing samples of both mouse and human experiments. Gene set enrichment analysis (GSEA) was performed on the differentially expressed genes using the H (hallmark) dataset from the MsigDB. The metric for ranking genes was set to Signal2Noise, all other parameters were as standard. Plots were generated using the Normalised Enrichment Score and the nominal *P* value.

### RNA isolation and RT-qPCR

Total RNA was extracted from cells using ReliaPrep (Promega). Reverse transcription was performed with the Tetro cDNA synthesis kit (Meridian) using Random Hexamers. qPCR was performed with Power SYBR green master mix (Applied Biosystems) in triplicates using the QuantStudio 5 Real-Time PCR System (ThermoFisher). Data were normalised against loading control. Primers used: *BAP1-FW 5‘-CGATCCATTTGAACAGGAAGA-3’*, *BAP1-REV 5‘-CTCGTGGAAGATTTCGGTGT-3’*, *ATM-FW 5’-CCGAGTGCAGTGACAGTGAT-3’, ATM-REV 5’-TTGACGGCAGCAGATAAGCA-3’, E2F1-FW 5’-CATCAGTACCTGGCCGAGAG-3’, E2F1-REV 5’-CCCGGGGATTTCACACCTTT-3’, GAPDH-FW 5’-GTCTCCTCTGACTTCAACAGCG-3’, GAPDH-REV 5’-ACCACCCTGTTGCTGTAGCCAA-3’*, *HPRT-FW 5’-GACACTGGCAAAACAATGCAGAC-3’, HPRT-REV 5’-TGGCTTATATCCAACACTTCGTGG-3’*.

### Animal studies

All animal procedures were performed in accordance with Dutch law and the institutional committees (Animal experimental committee and Animal welfare body) overseeing animal experiments at the Netherlands Cancer Institute, Amsterdam. Mice were housed under standard feeding, light cycles, and temperature with ad libitum access to food and water. All mice were housed in disposable cages in the laboratory animal centre (LAC) of the NKI, minimising the risk of cross-infection, improving ergonomics and obviating the need for a robotics infrastructure for cage-washing. The mice were kept under specific pathogen-free (SPF) conditions.

To establish xenografts, 5 × 10^6^ human mesothelioma cells in 100 μl PBS with 50% Matrigel (Corning) were subcutaneously implanted into the flank of 6–10-week-old NOD-Scid IL2Rγnull (NSG) male and female mice (Jackson Laboratory). Tumour growth was monitored by slide calliper 3 times a week (volume = length ×  width^2^/2). Tumours were allowed to grow to ~220 mm^3^ in size before randomisation into control and treatment groups, mice with tumours smaller than 220 mm^3^ 2 months after injection were excluded from the experiment. Randomisation was done by random distribution of experimental groups across multiple cages. Blinding was achieved as the experiment was performed by two independent persons from the in-house Intervention Unit. The person measuring the tumour volume and administering the drugs did not know the cage label and received the mice from the person recording mice weight. Mice were treated for 28 days and sacrificed after this time period. AZD1390 was administered intraperitoneally every day at 15 mg/kg, vehicle for this drug was Cremophor:DMSO:Water (1:1:8). GSK126 was administered once daily intraperitoneally at 30 mg/kg, vehicle for this drug was Captisol 20%. Mouse body weight was monitored every day. Mice were excluded from analysis if they were found dead in cage during the experiment.

### Quantification and statistical analysis

All statistical tests were performed using GraphPad Prism v.9 and R. Statistical significance was denoted as *P < 0.05, **P < 0.01, ***P < 0.001, and ****P < 0.0001. The number of independent experiments, samples, and type of statistical test are indicated in the figure legends. No statistical method was used to predetermine the sample size. In vivo data were compared by multiple unpaired two-sided Student’s *t* test when data were normally distributed.

## Results

### A focused drug synergy screen reveals potential synergistic partners with EZH2 inhibition

There is an extensive repertoire of inhibitors under preclinical investigation and a wide range of compounds that have been or are being used in clinical trials. To evaluate whether any of these compounds harbour combinatorial potential, we performed a focused drug synergy screen using EZH2 inhibition as an anchor. A panel of 20 drugs targeting key oncogenic pathways was tested (Table 1). We determined whether we could identify any synergistic effects according to the *Bliss independence model* [33].

**Table 1:** List of drugs and their clinical status used in the focused drug synergy screen.

Drug name	Target	Clinical stage	Indication	Targeted pathway or process
Palbociclib	CDK4/6	Clinically available	HR + HER2- breast cancer	Cell cycle checkpoint
MK-1775	WEE-1	Phase II	Trials running in multiple cancers	G2 DNA damage
NSC663284	CDC25	Pre-clinical	–	Cell cycle checkpoint
THZ1	CDK7	Pre-clinical	–	Cell cycle checkpoint
VE-822	ATR	Phase II	Trials running in multiple cancers	Genome integrity
AZD1390	ATM	Phase I	Brain cancer, solid tumours, non-small cell lung cancer	Genome integrity
Sonidegib	Hedgehog	Clinically available	Advanced Basal Cell Carcinoma	Hedgehog signalling
PF477736	CHK1	Phase I	Advanced solid tumours, terminated	Genome integrity
JQ1	BRD4	Pre-clinical	–	Chromatin
Nutlin3a	P53/MDM2	Phase I	Haematologic neoplasia	P53 pathway
Venetoclax	BCL2	Clinically available	Chronic Lymphocytic Leukaemia / Acute Myeloid Leukaemia	Apoptosis
Verteporfin	TEAD/YAP	Clinically available	Macular degeneration	Hippo signalling
Crizotinib	ALK/C-MET	Clinically available	Metastatic non-small cell lung cancer, anaplastic large cell lymphoma, inflammatory myofibroblastic tumour	RTK signalling
Panobinostat	HDAC	Clinically available	Multiple Myeloma	Chromatin
BI-2536	PLK1,2,3	Phase II	Metastatic carcinoma, uveal melanoma	Mitosis
SGI-1027	DNMT	Pre-clinical	–	Chromatin
TH287	MTH1	Pre-clinical	–	Cytoskeleton
Docetaxel	Microtubule	Clinically available	Approved for a wide range of cancers	Cytoskeleton
Cisplatin	DNA synthesis	Clinically available	Approved for a wide range of cancers	DNA replication

**Fig. 1:**
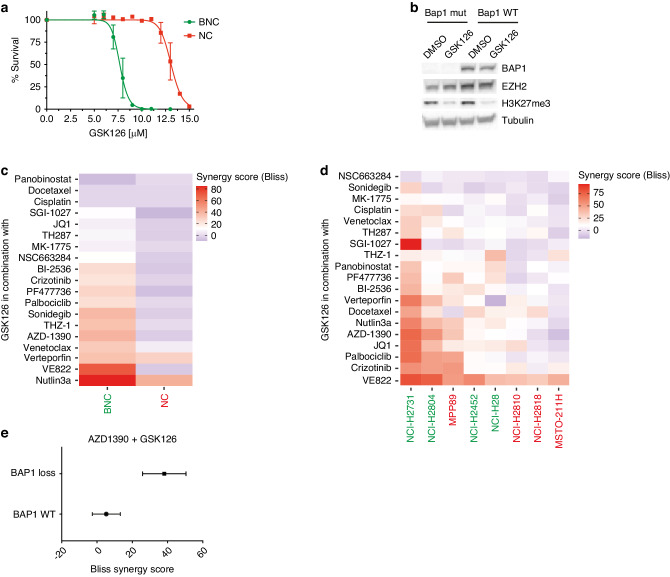
A small focused drug synergy screen reveals potential synergistic partners with EZH2 inhibition. **a** IC_50_ curves of Bap1-deficient (BNC) and Bap1-proficient (NC) mouse mesothelioma cell lines, IC_50_ ± SD are 7.7 μM ± 0.1 and 13.1 μM ± 0.2, respectively. **b** Western blot showing target inhibition of H3K27me3 after inhibiting EZH2 methyltransferase activity after 48 h of treatment with GSK126 in NC (Bap1 WT) and BNC (Bap1 mut) cell lines. Tubulin was used as a loading control. **c** Heatmap showing all the synergy scores obtained by the Bliss independence synergy model in mouse mesothelioma cell lines after 72 h of treatment with the combination. Drugs are ordered based on highest synergy average between the two cell lines. **d** Heatmap showing all the synergy scores obtained by the Bliss independence synergy model in human mesothelioma cell lines after 72 h of treatment with the combination. Drugs and cell lines are ordered based on highest synergy average between the cell lines and highest sensitivity, respectively. BAP1-deficient cell line names are shown in green, BAP1-proficient cell line names in red. **e** Plot showing the synergy scores of the combination with the ATM inhibitor, AZD1390. Plotted are the synergy scores of all the tested cell lines, both human and mouse, grouped on their BAP1 status.

Previously in our lab, we derived cell lines from our autochthonous mesothelioma mouse model with genetically defined BNC (*Bap1−/−, Nf2−/−, Cdkn2ab−/−*) or NC (*Nf2−/−, Cdkn2ab−/−*) background [21]. These mouse cell lines can serve as an excellent drug discovery platform based on BAP1 status as these cell lines have a similar genetic background. Determining the dose–response curves for EZH2 inhibition with GSK126 in these cell lines, shows a clear Bap1-status-specific shift in sensitivity (Fig. 1a). Target inhibition of the EZH2 methyltransferase activity was assessed, showing reduced levels of H3K27me3 level by western blot in both Bap1-proficient as well as Bap1-deficient cell lines (Fig. 1b). Using these mouse mesothelioma cell lines, we performed a 72h viability screen using 20 drugs and the EZH2 inhibitor GSK126 as anchor drug. Based on cell viability after treatment with single compounds, combinations with EZH2i, or controls a synergy score was calculated using SynergyFinder [31, 32]. Drug combination effect was assessed using the Bliss independence model. According to this model, if the effect of the combination is greater than the additive effect of the single drugs the response can be classified as synergistic (Bliss Score >10), antagonism is indicated if the combined effect is less than the expected effect (Bliss Score < −10), any scores in between these values are considered non-interactive. All the calculated scores were incorporated in a heatmap and ordered by highest synergy score (Fig. 1c).

To increase the clinical relevance and to validate the findings from our mouse cell lines, we performed a similar screen in 8 human malignant mesothelioma cell lines (4 BAP1 mutant, 4 BAP1 wild-type) (Supplementary Fig. 1a). Results were again assessed using the Bliss independence model and the scores were put in a heatmap and ordered for highest synergy score and cell line sensitivity (Fig. 1d). Interestingly, we see that the included ATM inhibitor AZD1390, shows high synergistic scores in Bap1-deficient mouse mesothelioma cell lines and in human cell lines almost exclusively in BAP1-deficient cells (Fig. 1d, e). Remarkably, VE- 822, an ATR inhibitor, showed potential synergy in Bap1-deficient but not in Bap1-proficient mouse cells. Notably, it showed high synergy scores in all human cell lines. However, we were not able to validate these results in clonogenicity assays, which might be due to the high efficacy of single VE-822 treatment potentially indicating toxicity (Supplementary Fig. 1b). Furthermore, we also observe synergistic potential for combinations with Palbociclib, an CDK4/6 inhibitor, and Crizotinib, an inhibitor of c-MET and ALK. As these were not as pronounced as observed with the ATM inhibitor, AZD1390, and given the published evidence for the potential involvement of BAP1 in double-strand break (DSB) repair we focus on this combination [35, 36].

### Combining EZH2 and ATM inhibition shows high synergistic potential in BAP1-deficient mesothelioma

**Fig. 2:**
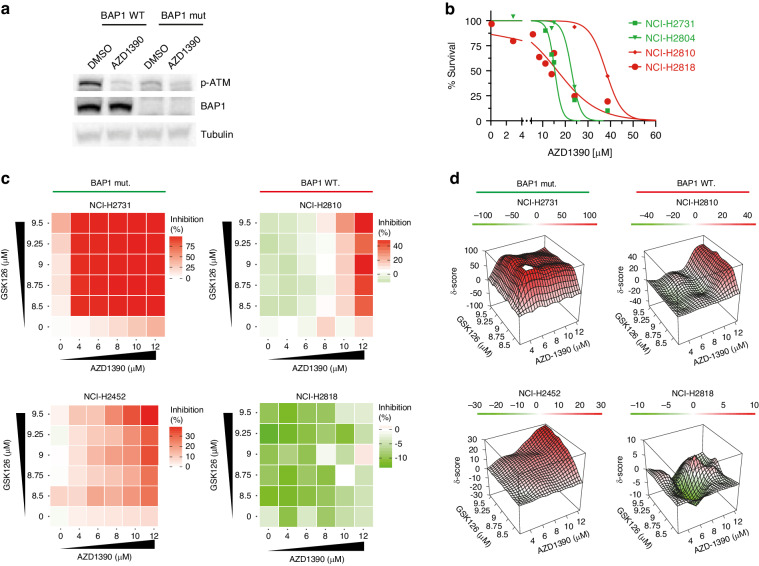
Combining EZH2 and ATM inhibition shows high synergistic potential in BAP1-deficient mesothelioma. **a** Western blots showing target inhibition of p-ATM after 48 h of treatment with AZD1390 in a BAP1 mutant and a BAP1 wild-type human mesothelioma cell line. Tubulin served as loading control. **b** IC_50_ curves of BAP1-deficient (H2731, H2804) and BAP1-proficient (H2810, H2818) mouse mesothelioma cell lines, IC_50_ ± SD are 15 μM ± 2, 23 μM ± 2, 38 μM ± 3, and 16 μM ± 2 respectively. **c** Heatmaps generated by the SynergyFinder webtool, showing the percentage of inhibition of single treatment and combination treatment with GSK126 and AZD1390. Inhibition percentages are indicated by a green/red scale, red for increasing inhibition. **d** 3D plots generated by the SynergyFinder tool, showing all the individual synergy scores for the single and combination treatments with GSK126 and AZD1390. The Bliss independence score (δ) is indicated on the *y* axis and drug concentrations on *x* and *z* axis.

First, target inhibition of ATM activity was assessed, showing reduced levels of p-ATM by western blot in both BAP1-proficient as BAP1-deficient cell lines (Fig. 2a). We determined dose–response curves of ATM inhibition for the tested human mesothelioma cell lines showing that there is no clear difference in sensitivity between Bap1-deficient and proficient cell lines for ATM inhibition only (Fig. 2b). To further explore the potential synergy of the combined inhibition of EZH2 and ATM, we zoomed in on the obtained synergy scores. Heatmaps visualised by SynergyFinder web application tool clearly show that for BAP1-deficient cell lines (NCI-H2731 and NCI-H2804) low dosages of drug combination (lower-left corner of heatmap) exert a big increase in inhibition as compared to single drug dosage. Conversely, the results for BAP1-proficient cell lines (NCI-H2810 and NCI-H2818) show very little effect even at high concentrations (Fig. 2c and Supplementary Fig. 2a). Visualising the synergistic effects of these compounds in 3D plots show a strong image of synergy over a robust concentration range in BAP1-deficient cell lines only (Fig. 2d and Supplementary Fig. 2b). To ratify our findings, similar experiments were done with another ATM inhibitor, KU-60019. Experiments using this inhibitor also showed a clear BAP1-status-specific synergistic effect (Supplementary Fig. 3a–d).

### The observed synergy of the combination can be validated in in vitro models

**Fig. 3:**
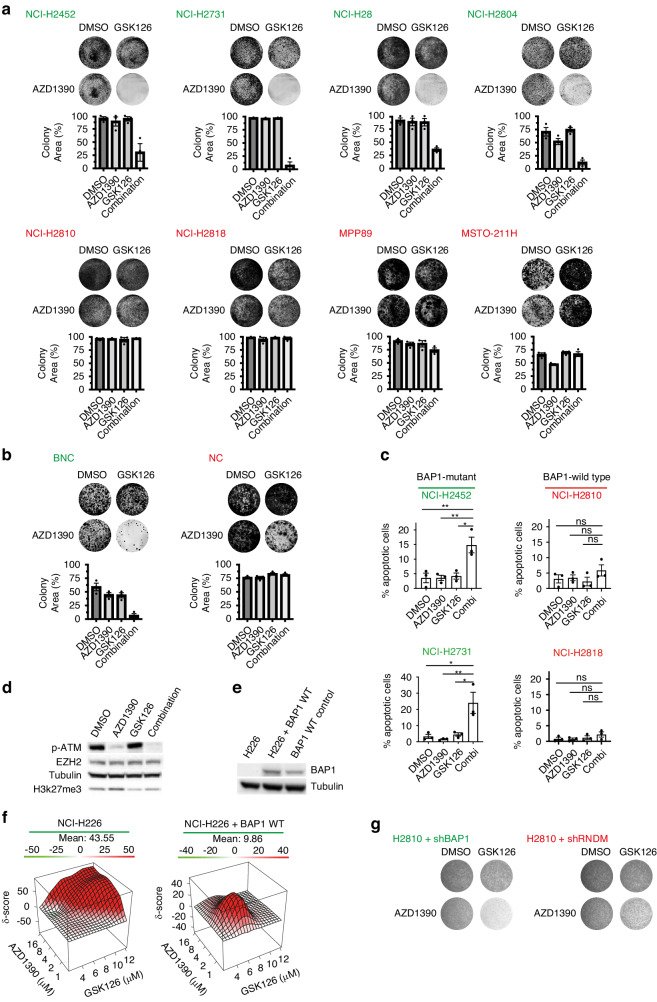
Observed synergy of the combination can be validated in in vitro models. **a** Colony-formation assays and quantifications showing sensitivity of BAP1-deficient human mesothelioma cell lines to combination treatment with 1 μM AZD1390 (ATMi) and 7.25 μM GSK126 (EZH2i) and insensitivity of BAP1-proficient cell lines; deficient cell lines are indicated in green, proficient cell lines in red, representative data shown from three independent experiments. Quantification data are mean ± SEM, *n* = 3 independent experiments. **b** Likewise for mouse mesothelioma cell lines. **c** Apoptosis assays using Annexin V-FITC-PI staining shows increased amounts of apoptotic cells in BAP1-mutant cell lines upon combination treatment. Conversely, this is not seen for BAP1 wild-type cell lines; 3 μM AZD1390, 6.25 μM GSK126, data shown is mean ± SEM, *n* = 3 independent experiments. **d** Western blots showing target inhibition for single and combination treatment after 48 h. **e** Western blot showing successful re-expression of BAP1 protein in NCI-H226 cells. As control NCI-H2810, a known BAP1 wild-type line, was used. **f** 3D plots generated by the SynergyFinder tool, showing synergy scores of BAP1-deficient NCI-H226 and its BAP1-proficient counterpart for the combination treatments with GSK126 and AZD1390. The Bliss independence score (δ) is indicated on the y axis and drug concentrations on *x* and *z* axis. **g** Clonogenicity assay showing the increased sensitivity to the combination treatment with 1 μM AZD1390 (ATMi) and 7.25 μM GSK126 (EZH2i) upon depletion of BAP1 via inducible shBAP1 construct. Representative data shown from three independent experiments.

To further corroborate on our finding of the possible synergistic interaction between EZH2i with ATMi, we performed long-term clonogenicity assays. First, we used our human mesothelioma cell line panel to validate the BAP1-status-specific efficacy of the drug combination on cell survival. Cells were treated with GSK126 (7.25 μM), AZD1390 (1 μM), or the combination of both. In BAP1-mutant cells the treatment with the combination showed high efficacy in all four BAP1 negative lines, whereas single compound treatment had no or limited effect on these cells. In contrast, single or combinatorial compound treatment had no or little effect on the BAP1-proficient cell lines (Fig. 3a). A similar observation was made for mouse mesothelioma cell lines (Fig. 3b). Experiments performed with the alternative ATM inhibitor KU-60019 gave similar results showing a sensitivity exclusively in BAP1-deficient cells (Supplementary Fig. 4a, b). Similarly, interchanging the EZH2 inhibitor GSK126 with Tazemetostat showed efficacy against BAP1-deficient cells (Supplementary Fig. 4c). In addition, apoptosis assays were performed 48 h after drug treatment. In line with our expectations, we observed a significant increase in apoptotic cells upon combination treatment in BAP1-deficient cell lines but not in BAP1-proficient cell lines (Fig. 3c). Target inhibition was validated by western blot (Fig. 3d). In order to verify whether the efficacy of our combination can be truly attributed to the absence or presence of BAP1 protein expression we used the isogenic mesothelioma cell line NCI-H226. NCI-H226 cells are homozygously deleted for BAP1 and have a complete loss of BAP1 expression, in its isogenic counterpart a wild-type BAP1 vector is stably expressed (Fig. 3e). Upon treatment with EZH2i combined with a concentration range of ATM inhibitor we observed a decrease in synergy score of the combination in the BAP1 WT expressing variant confirming the BAP1-status-specific sensitivity of the combination (Fig. 3f). In addition, we performed clonogenicity assays in a BAP1-proficient cell line with an inducible shBAP1 construct and observe that upon induction of this construct these cells become more sensitive to the combination (Fig. 3g). Collectively, our data shows that the combinatorial strategy of EZH2i and ATMi is a highly efficacious treatment against BAP1-deficient mesothelioma in vitro.

### The combination of ATM inhibition and EZH2 inhibition limits tumour growth in BAP1-deficient human xenografts

**Fig. 4:**
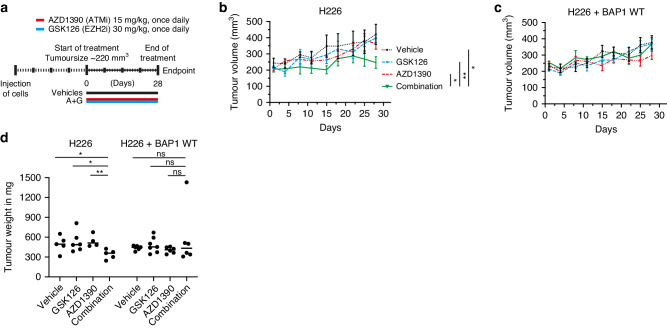
The combination of ATM inhibition and EZH2 inhibition limits tumour growth in BAP1-deficient human xenografts. **a** Schematic representation of the treatment schedule of the tumour-bearing mice. NSG mice were treated with a combination of AZD1390 (ATMi) and GSK126 (EZH2i) for 28 consecutive days. **b** NSG mice with NCI-H226 BAP1-deficient xenografts were treated with vehicle, AZD1390 (15 mg/kg once daily), GSK126 (30 mg/kg once daily), or a combination. Shown is mean tumour volume over time (tumour volume ± SEM; *n* = 5–7 mice per treatment group). **c** Likewise for NSG mice with NCI-H226 + BAP1 WT xenografts. **d** Tumour weight of the mice with NCI-H226 xenografts and the isogenic BAP1 WT counterpart in the cohorts, tumours were weighed directly after dissection at the end of the treatment period. *P* values were determined by two-tailed unpaired Student’s *t* test; *P < 0.05, **P < 0.01.

To test the efficacy of our drug combination in vivo, we transplanted the human mesothelioma cell lines NCI-H226 and its isogenic counterpart NCI-H226 + BAP1 WT into NOD-Scid IL2Rγnull mice. Tumours were allowed to form, and mice (*n* = 5–7) bearing tumours of ~220 mm^3^ were treated for 28 days with the EZH2 inhibitor, GSK126, and the ATM inhibitor, AZD1390, or the combination of both drugs (Fig. 4a). Tumour volume was monitored over time and tumour weights were analysed at the end of the experiment. We observed that, in contrast to single-agent treatment, the combination of GSK126 and AZD1390 resulted in significant growth inhibition of the BAP1-deficient NCI-H226 xenografts compared to its BAP1-proficient counterpart (Fig. 4b, c). In addition, tumour weight of BAP1-deficient xenografts treated with the combination were also lower as compared to single-agent-treated xenografts. In BAP1-proficient xenografts no statistically significant differences were observed (Fig. 4d). These in vivo results thus show the therapeutic potential of the proposed combination for BAP1-deficient mesothelioma.

### Acute BAP1 loss leads to a consequent reduction in ATM levels

**Fig. 5:**
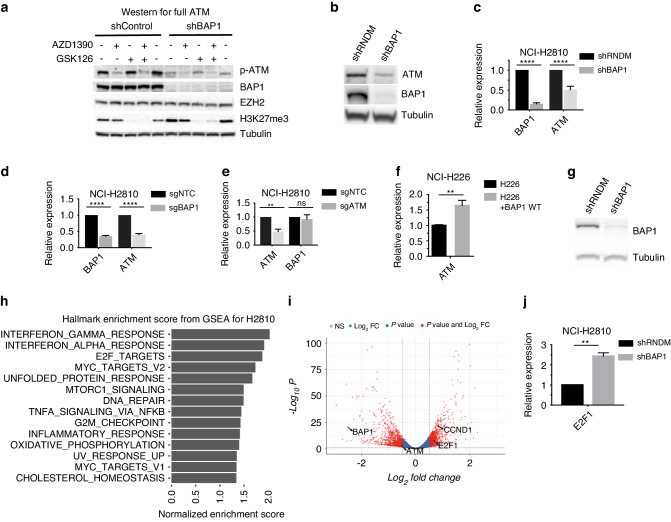
Acute BAP1 loss leads to consequent reduction in ATM levels. **a** Western blot for analysis of target inhibition in inducible shBAP1 human mesothelioma cell line NCI-H2810 suggests lower overall expression of p-ATM protein. **b** Western blot shows lower total ATM levels upon depletion of BAP1 with inducible shBAP1 construct. **c** qPCR validation of lowered expression of ATM in BAP1 depleted human mesothelioma cell lines with inducible shBAP construct, 48 h after induction with doxycycline. Values are normalised to induced shRANDOM control samples; mean ± s.d.; *n* = 3 independent experiments. **d** Likewise for NCI-H2810 with synthetic guide RNA against BAP1 or non-targeting control; mean  ± s.d.; *n* = 3 independent experiments. **e** qPCR validation of stable BAP1 levels in ATM depleted cells via synthetic guide RNA. Values are normalised to sgRNA non-targeting control samples; mean ± s.d.; *n* = 3 independent experiments. **f** Re-expression of BAP1 in NCI-H226 cell line shows an increase in ATM levels on qPCR; mean ± s.d.; *n* = 3 independent experiments. **g** Western blot validation of BAP1 depletion via inducible shBAP1 construct, 72 h after induction with doxycycline in NCI-H2810 samples send for RNA sequencing. **h** Bar plot of pathway enrichment of hallmark gene sets within the MSigDB upon shBAP1 induction in NCI-H2810 cells; shown are the pathways with FDR < 0.25 and *P* < 0.05. **i** Volcano plot representing the changes in expression of genes with *P* < 0.05, Log2FoldChange > |0.5 | , shown only are the genes differentially expressed in both NCI-H2810 and MSTO-211H with a published link to ATM transcriptional regulation. **j** qPCR validation of increased E2F1 levels upon induction of shBAP1 construct, 48 h after induction; mean ± s.d.; *n* = 3 independent experiments.

Upon drug target validation with western blot in cells with an inducible shBAP1 construct, we observed that upon induction of the shRNA construct with doxycycline, the phospho-ATM levels were reduced in comparison to cells with the control construct (Fig. 5a). In addition, total ATM protein levels were also low in these acute BAP1 depleted cells. In contrast we do not observe consistently lower (p-)ATM levels in the BAP1-deficient tumour cell lines (Fig. 5b and Supplementary Fig. 5a). To see whether this correlated with mRNA levels we performed RT-qPCR on these BAP1 knock-down cells and observe that in addition to protein levels, mRNA levels of ATM are also lower (Fig. 5c). A similar effect was seen in other cell lines with the same construct (Supplementary Fig. 5b). To validate our findings, we repeated these experiments using a synthetic guide against BAP1 and observed lower ATM mRNA levels upon BAP1 deletion (Fig. 5d and Supplementary Fig. 5c). In contrast, knocking-out ATM with a similar method had no effect on BAP1 mRNA levels suggesting that while BAP1 is able to regulate ATM levels, the reverse is not true (Fig. 5e and Supplementary Fig. 5d). Using the previously described NCI-H226 cell line we observe that re-expressing BAP1 protein leads to subsequent upregulation of ATM expression, further corroborating our findings in the shBAP1 cell lines (Fig. 5f). To get more insight in the potential link between BAP1 loss and reduction in ATM levels, we performed RNA sequencing on the mesothelioma cell lines with an inducible shBAP1 construct and compared these lines to cells transduced with an inducible shRANDOM construct. shRNA constructs were induced with doxycycline for 48 h and harvested for RNA sequencing (Fig. 5g). Initial gene set enrichment analysis (GSEA) for Hallmark genesets on our data shows enrichment for the DNA repair hallmark, in line with published literature linking BAP1 to DNA damage pathways (Fig. 5h and Supplementary Fig. 5e). In addition, we find gene set enrichment for both the G2M checkpoint hallmark as well as for E2F target hallmark gene set. Further, we analysed the genes (*n* = 15) that are possibly involved in this process and are linked to transcriptional regulation of ATM. We filtered this gene list from our RNA sequencing dataset. Only genes were considered that have an adjusted *P* value < 0.05 and a Log2FoldChange of > |0.5 | . Surprisingly, only E2F1 and CCND1 are significantly differentially expressed in both our cell lines upon knock-down of BAP1 (Fig. 5i and Supplementary Fig. 5f). Validation of the change in expression of these genes by RT-qPCR showed a significant upregulation of E2F1 expression only, additionally, western blots show a similar trend for E2F1 levels (Fig. 5j and Supplementary Fig. 5g). E2F1 has known links to both ATM and BAP1 [37–39]. These data suggest that there might be indirect regulation of ATM expression by BAP1 via E2F1.

## Discussion

Treatment options for malignant mesothelioma are limited and inevitably all unresected or recurrent patients die of this disease. Over the past years multiple studies and clinical trials have been executed, with the recently approved immune checkpoint blockade therapy as the most successful one [6, 24, 40]. However, there is still a big fraction of patients that do not respond to this treatment. Where other cancers largely benefit from precision targeting this field is not widely explored in mesothelioma [41]. Adding to the difficulty of developing novel treatments is that the most common alterations in mesothelioma are the inactivation of tumour suppressor genes [42, 43]. In this study, by performing combination drug screens we exploit BAP1 as a potential stratification biomarker for a highly synergistic combination targeting ATM and EZH2 simultaneously.

BAP1 is one of the most frequently altered genes in mesothelioma patients. Our findings demonstrate that these BAP1 alterations can serve as an interesting target to base novel treatment modalities on. Previously it has been shown that cells deficient for BAP1 are sensitive to EZH2 inhibition [20]. However, recently published clinical trial data show that the efficacy of EZH2 inhibition as a single agent is very limited [23]. Besides the limited observed efficacy, tumours are also well-known to acquire resistance overtime to monotherapies paving the way for combination strategies [44, 45]. Studies in other malignancies adding a second compound on top of EZH2 inhibition show that inhibiting EZH2 can lead to novel sensitivities [27–29, 46–48]. Therefore, using EZH2 inhibition as an anchor and complementing it with a second inhibitor could lead to improved treatment strategies.

ATM plays a major role in normal cells protecting the genome against DNA damage by responding to DNA double-strand breaks and other lesions minimising mutations risks potentially leading to cancerous cells. In cancerous cells the same function of ATM might favour tumour growth and cancer cell survival. Therefore, in recent years multiple specific inhibitors have been developed to target ATM of which some are currently in Phase I clinical trials. One such compound is AZD1390 that has been tested preclinically in combination with Olaparib against glioblastoma [49]. Our extensive testing in preclinical models of BAP1-deficient mesothelioma show that inhibiting ATM in combination with EZH2 is highly synergistic, showing the potential therapeutic application of this combination. In addition, our clonogenicity assays demonstrate that lower drug dosages are needed in combination to limit cell proliferation compared to single-agent treatment, likely limiting toxicity risks for patients. Strengthening our observations is a previous publication showing synthetic lethality of this combination in BRCA1-deficient breast cancer [26].

Besides the extensively described combination of EZH2 inhibitor with ATM inhibitors we also observed other potentially synergistic hits that are worthy of further investigation. For example, verteporfin, an inhibitor of YAP/TAZ-TEAD, showed synergy in both our Bap1-deficient and Bap1-proficient mouse cell lines. This seems logical as these cell lines are derived from mice that are *Nf2* negative and *NF2* mutations have been shown to promote sensitivity to inhibition of YAP [50]. It will be interesting to further investigate this combination in the context of NF2 mutations. Another compound showing high synergy scores was Nutlin3a which inhibits the interaction between p53/MDM2. Disruption of this interaction leads to the release of p53 and transcription of its target genes engaging in remaining DNA integrity. It is therefore not surprising that we see efficacy of this compound in our mouse cell lines that are p53 wild- type [51]. As the TP53 gene is mutated in only 8–19% of mesothelioma patients it will be interesting to further investigate this combination in TP53 wild-type tumours [52]. Further, we observe some inconsistencies in synergy scores between mouse and human cell lines. These variations can be explained by the fact that our mouse mesothelioma cell lines are genetically similar except for their Bap1 status whereas the human tumour-derived cell lines have co-mutations which might affect the efficacy of the combinations. This may be why some combinations that are synergistic in mouse cell lines are not necessarily synergistic in human cell lines.

BAP1, was originally identified as a nuclear protein shown to bind the RING finger domain of BRCA1, consecutive studies suggested that instead of BRCA1 BAP1 binds to BARD1 [53, 54]. Additional studies have shown that catalytic BAP1 activity is important for DSB DNA repair (DDR) by homologous recombination however the way in which BAP1 does this remains largely unknown [35, 36]. As reports suggest that BAP1 is able to regulate the expression of DDR protein encoding genes, indirect regulation of DSB repair proteins like ATM by BAP1 via gene expression regulation could be an explanation [13, 55]. In line with that hypothesis, in the current study we show that the acute loss of BAP1 in mesothelioma cells leads to the subsequent loss of ATM expression at both mRNA and protein level. Notably, such differences in ATM levels were not seen between BAP1-proficient and deficient cell lines, suggesting a possible compensatory mechanism to circumvent ATM repression in in vitro culture; however, this needs further investigation. RNA sequencing performed on cell lines with an inducible shRNA against BAP1 showed that the well-known transcription factor E2F1 was upregulated upon acute depletion of BAP1. BAP1 and E2F1 might be linked via the p16/Rb, a known negative regulator of E2F proteins, it has previously been shown that Polycomb complexes PRC1 and PRC2 are able to repress p16 [56, 57]. Therefore, it is likely that in case of mesothelioma E2F1 expression gets activated by genetic deletion of p16 or by PRC2-mediated repression. The observed upregulation of E2F1 might influence the levels of ATM as its promoter region, shared with NPAT, has up to five E2F protein binding sites [38]. Furthermore, a study in prostate cancer demonstrated that upon exposure to doxorubicin E2F1 was recruited to the promoter region of ATM and repressed its expression [39]. Together with our data, this suggests that lower ATM expression upon the loss of BAP1 might be, at least partially, due to transcriptional repression via E2F1.

## Conclusions

In summary, we demonstrate that the simultaneous inhibition of EZH2 and ATM is a highly synergistic regimen against preclinical models of BAP1-deficient mesothelioma. In addition, we show that expression of the major protein kinase ATM gets lowered upon the loss of BAP1, potentially due to transcriptional repression via E2F1. Taken together, all this data warrants further research into the indirect link between BAP1 and ATM and how this might affect DSB repair, and facilitates the investigation for clinical possibilities of this combination strategy against BAP1-deficient mesothelioma.

### Supplementary information

Supplementary Figures

## Data Availability

The datasets used and analysed during this study are available from the corresponding author on reasonable request.

## Code availability

The script that was used to analyse the data described above is available upon request.
