# Supplementary material for: Combination of EZH2 and ATM inhibition in BAP1-deficient mesothelioma
Source: Br J Cancer. 2024 Mar 22;130(11):1855–65. doi: 10.1038/s41416-024-02661-3 (PMC11130181; doi:10.1038/s41416-024-02661-3)
Supplement: Supplementary file 1 — Supplementary Figures [file 41416_2024_2661_MOESM1_ESM.pdf]

Supplementary Figure 1

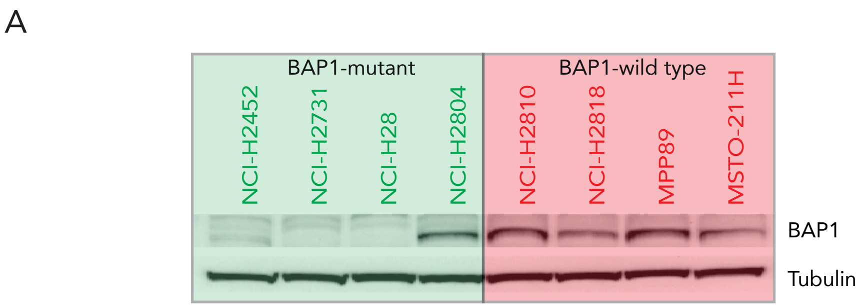


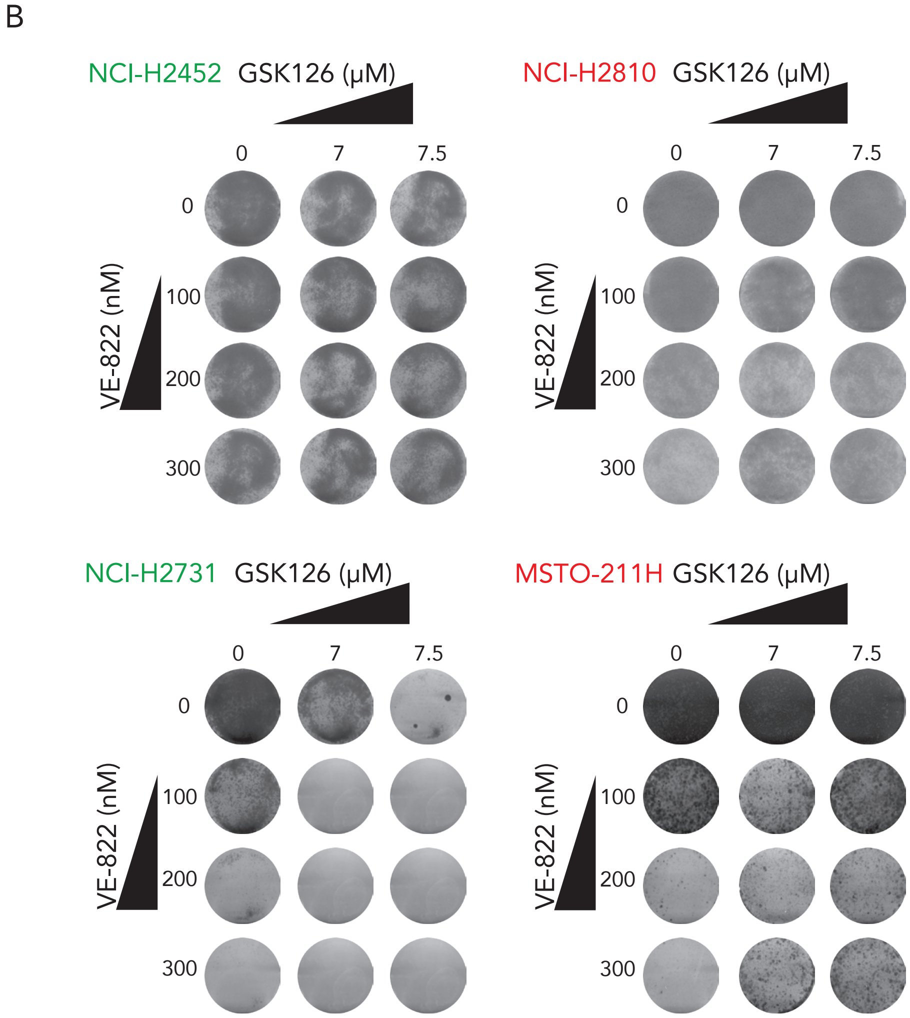


**Supplementary Figure 1. A small foussed drug synergy screen reveals potentia synergistic partners with EZH2 inhibition. A,** Western blot showing the BAP1 status of the used human mesothelioma cell lines. NCI-H2804 is a catalytically inactive BAP1 mutant thus showing BAP1 protein on the blot. Tubulin was used as loading control. **B,** Colony-formation assays showing that found synergy in focused drug screen cannot be validated in all the tested cell lines for the combination of VE-822 (ATRi) and GSK126 (EZH2i); BAP1-deficient cell lines are indicated in green, BAP1-proficient cell lines in red, representative data shown from three independent experiments.

Supplementary Figure 2

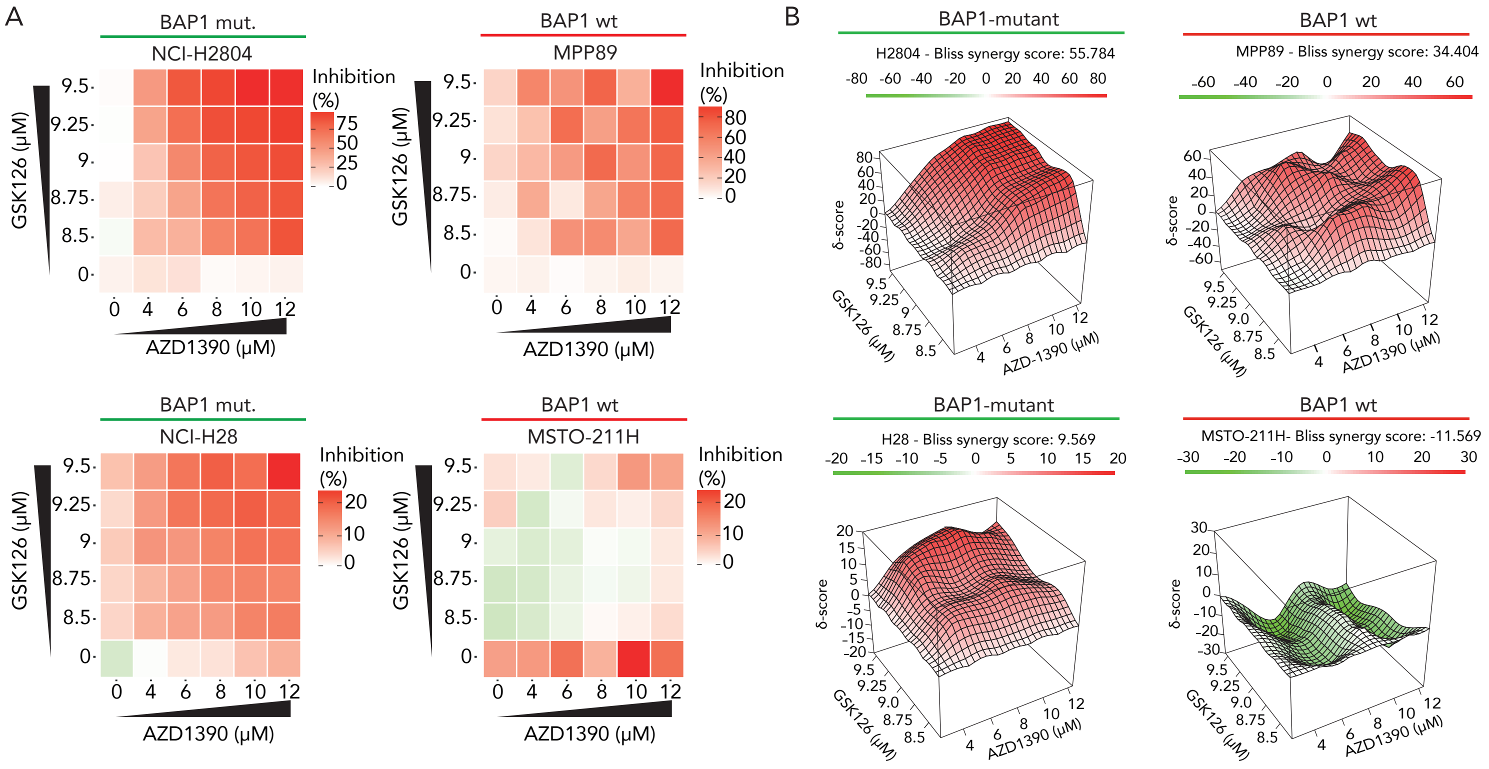


**Supplementary Figure 2. Combining EZH2 inhibition and the ATM inhibitor AZD1390 shows high synergistic potential in BAP1-deficient mesothelioma. A,** Heatmaps generated by the SynergyFinder webtool, showing the percentage of inhibition of single treatment and combination treatment with GSK126 and AZD1390 in additional human mesothelioma cell lines. Inhibition percentages are indicated by a green/red scale, red for increasing inhibition. **B,** 3D plots generated by the SynergyFinder tool, showing all the individual synergy scores for the single and combination treatments with GSK126 and AZD1390 in additional human mesothelioma cell lines. The Bliss independence score (δ) is indicated on the y-axis and drug concentrations on x- and z-axis.

Supplementary Figure 3

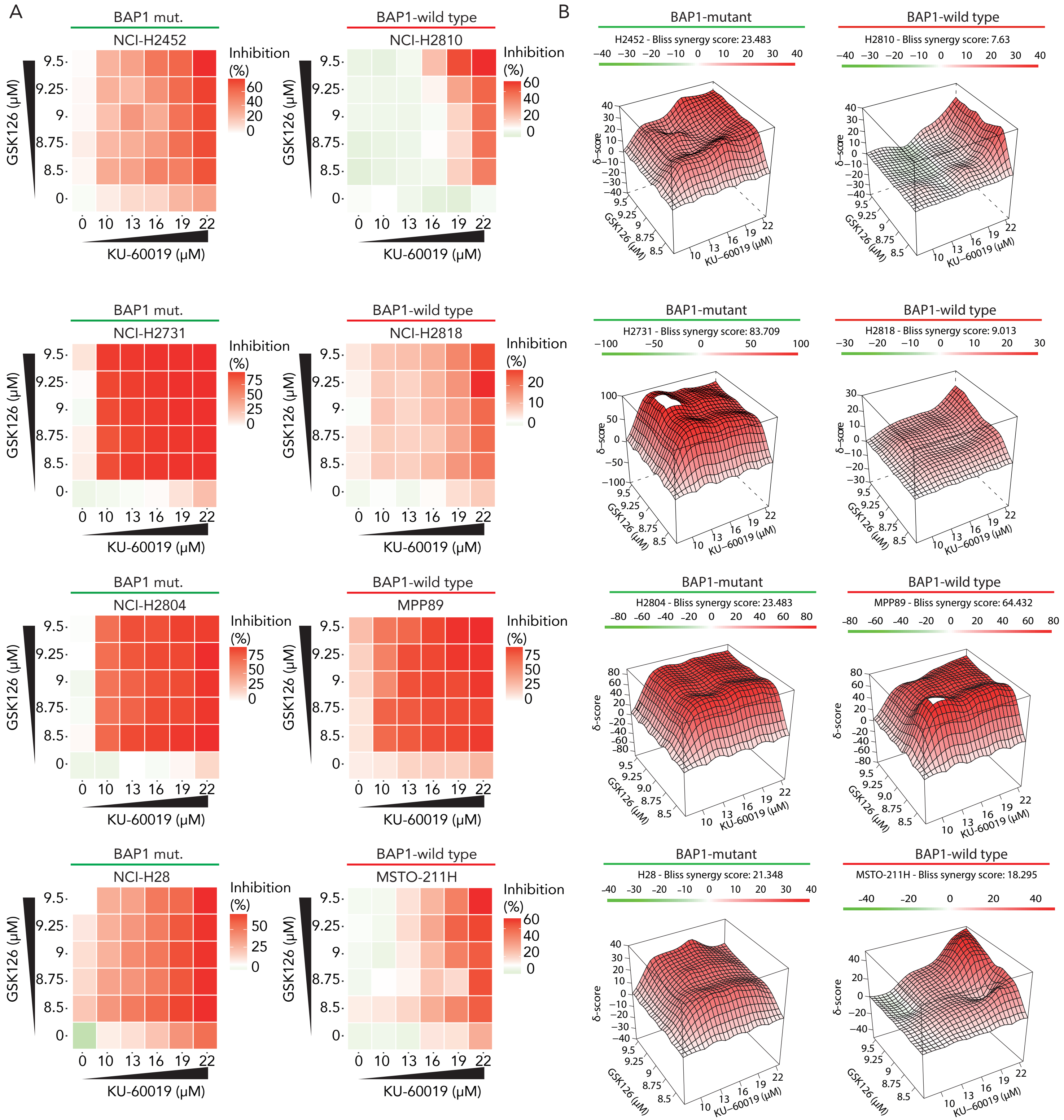

**Supplementary Figure 3. Combining EZH2 inhibition and an alternative ATM inhibitor shows high synergistic potential in BAP1-deficient mesothelioma. A,** Heatmaps generated by the SynergyFinder webtool, showing the percentage of inhibition of single treatment and combination treatment with GSK126 and KU-60019 in BAP1-deficient and -proficient human mesothelioma cell lines. Inhibition percentages are indicated by a green/red scale, red for increasing inhibition. **B,** 3D plots generated by the SynergyFinder tool, showing all the individual synergy scores for the single and combination treatments with GSK126 and KU-60019 in BAP1-deficient and -proficient human mesothelioma cell lines. The Bliss independence score (δ) is indicated on the y-axis and drug concentrations on x- and z-axis.

Supplementary Figure 4

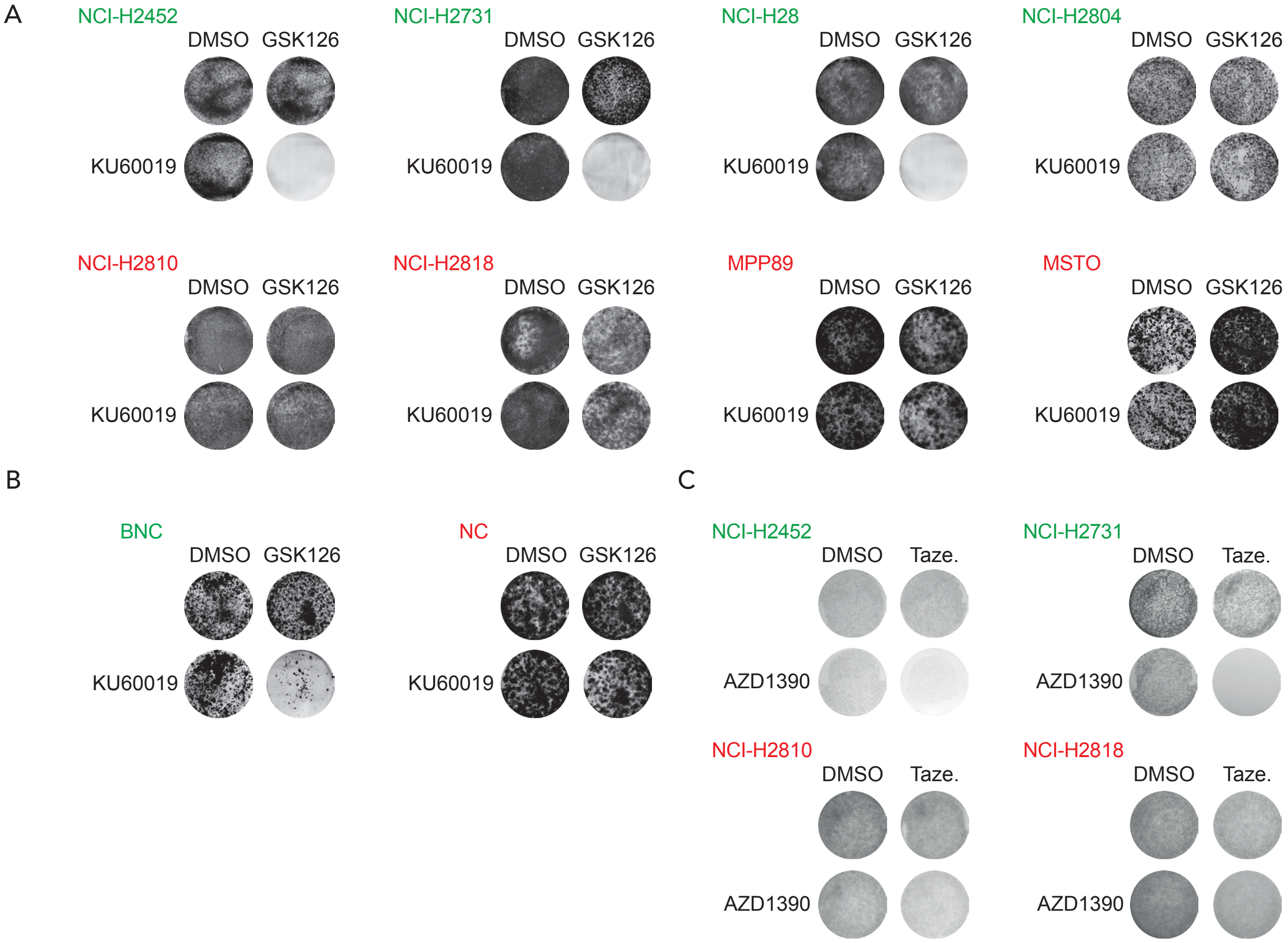


**Supplementary Figure 4. Observed synergy of the combination can be validated in in vitro models. A,** Colony-formation assays showing sensitivity of BAP1-deficient human mesothelioma cell lines to combination treatment with 1μM KU-60019 (ATMi) and 7.25μM GSK126 (EZH2i) and insensitivity of BAP1-proficient cell lines; deficient cell lines are indicated in green, proficient cell lines in red, representative data shown from three independent experiments. **B,** Likewise for mouse mesothelioma cell lines. **C,** Colony-formation assays showing sensitivity of BAP1-deficient human mesothelioma cell lines to combination treatment with 3μM AZD1390 (ATMi) and 15μM Tazemetostat (EZH2i) and insensitivity of BAP1-proficient cell lines; deficient cell lines are indicated in green, proficient cell lines in red, representative data shown from three independent experiments.

## Supplementary Figure 5

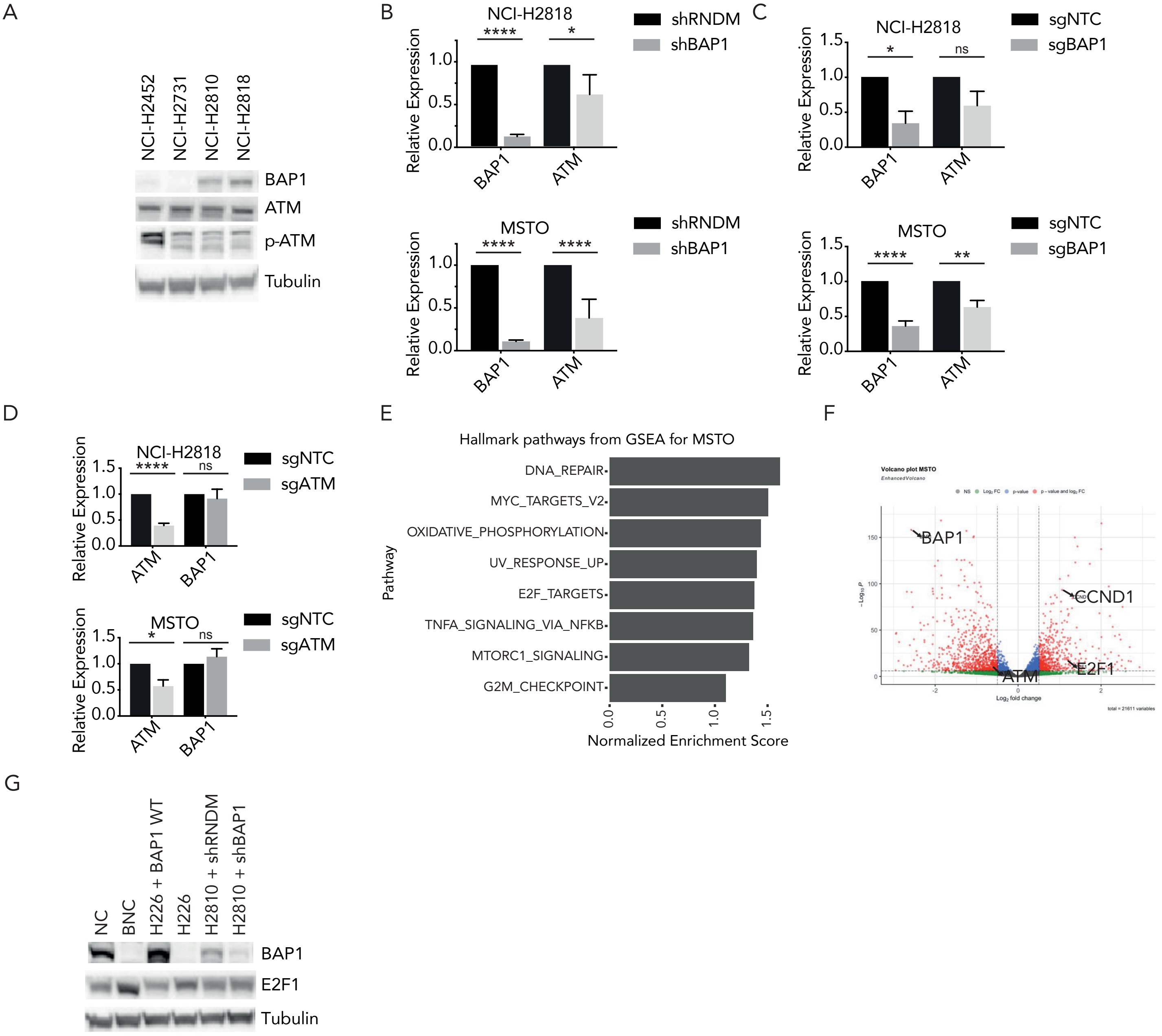


**Supplementary Figure 5. Acute BAP1 loss leads to consequent reduction in ATM levels. A,** Western blot of human tumour cell lines for BAP1 status and (p-)ATM protein levels. Tubulin was used as a loading control. **B,** qPCR validation of lowered expression of ATM in BAP1 depleted human mesothelioma cell lines NCI-H2818 and MSTO-211H with inducible shBAP construct, 48 hours after induction with doxycycline. Values are normalized to induced shRANDOM control samples; mean ± s.d.; n = 3 independent experiments. **C,** Likewise for NCI-H2818 and MSTO-211H with synthetic guide RNA against BAP1 versus non-targeting control; mean ± s.d.; n = 3 independent experiments. **D,** qPCR validation of stable BAP1 levels in ATM depleted cells via synthetic guide RNA. Values are normalized to sgRNA non-targeting control samples; mean ± s.d.; n = 3 independent experiments. **E,** Barplot of pathway enrichment of hallmark gene sets within the MSigDB upon shBAP1 induction in MSTO-211H cells; shown are the pathways with FDR <0.25 and $p < 0.05$. **F,** Volcano plot representing the changes in expression of genes with $p < 0.05$, Log2FoldChange > |0.5|, shown only are the genes differentially expressed in both NCI-H2810 and MSTO-211H with a published link to ATM transcriptional regulation. **G,** Western blot of mouse and human BAP1-deficient mesothelioma cell lines and their BAP1-proficient counterparts for BAP1 and E2F1 protein levels. Tubulin was used as a loading control.
